# Comparison of gene co-networks reveals the molecular mechanisms of the rice (*Oryza sativa L*.) response to *Rhizoctonia solani* AG1 IA infection

**DOI:** 10.1007/s10142-018-0607-y

**Published:** 2018-05-05

**Authors:** Jinfeng Zhang, Wenjuan Zhao, Rong Fu, Chenglin Fu, Lingxia Wang, Huainian Liu, Shuangcheng Li, Qiming Deng, Shiquan Wang, Jun Zhu, Yueyang Liang, Ping Li, Aiping Zheng

**Affiliations:** 1 Rice Research Institute of Sichuan Agricultural University, Chengdu, 611130 China; 2 State Key Laboratory of Hybrid Rice, Sichuan Agricultural University, Chengdu, 611130 China; 3 Key Laboratory of Sichuan Crop Major Disease, Sichuan Agricultural University, Chengdu, 611130 China

**Keywords:** Rice, *Rhizoctonia solani* AG1 IA, Transcriptomics, Co-expression network

## Abstract

*Rhizoctonia solani* causes rice sheath blight, an important disease affecting the growth of rice (*Oryza sativa* L.). Attempts to control the disease have met with little success. Based on transcriptional profiling, we previously identified more than 11,947 common differentially expressed genes (TPM > 10) between the rice genotypes TeQing and Lemont. In the current study, we extended these findings by focusing on an analysis of gene co-expression in response to *R. solani* AG1 IA and identified gene modules within the networks through weighted gene co-expression network analysis (WGCNA). We compared the different genes assigned to each module and the biological interpretations of gene co-expression networks at early and later modules in the two rice genotypes to reveal differential responses to AG1 IA. Our results show that different changes occurred in the two rice genotypes and that the modules in the two groups contain a number of candidate genes possibly involved in pathogenesis, such as the VQ protein. Furthermore, these gene co-expression networks provide comprehensive transcriptional information regarding gene expression in rice in response to AG1 IA. The co-expression networks derived from our data offer ideas for follow-up experimentation that will help advance our understanding of the translational regulation of rice gene expression changes in response to AG1 IA.

**Electronic supplementary material:**

The online version of this article (10.1007/s10142-018-0607-y) contains supplementary material, which is available to authorized users.

## Introduction

Rice sheath blight, which is caused by the soil-borne basidiomycete fungus *Rhizoctonia solani*, is an economically destructive and widespread disease throughout rice production areas worldwide (Zheng et al. 2013). As one of the most destructive diseases in cultivated rice (*Oryza sativa* L.), rice sheath blight caused by *R. solani* AG1 IA can result in yield losses of 5–10% and even 50% in severe cases (Shu et al. 2015). Accompanying cultivars that are susceptible to sheath blight are planted in most rice-growing regions of the world, which increases yield losses from this disease. Although rice varieties that are relatively resistant to sheath blight have been identified (Prasad and Eizenga 2008), no completely field resistant rice cultivar or immune variety has been found (Zeng et al. 2015; Taheri and Tarighi 2011). At present, management of this disease mainly involves chemical methods. Despite the importance of rice sheath blight, research in resistance has been slow, partly due to the lack of a standard resistance identification methods and because the disease is easily affected by in-field conditions on small-scale farmland (Wang et al. 2015a, b; Yugander et al. 2014). To date, few in-depth studies have investigated the molecular mechanism of rice sheath resistant during or after fungal entry into host tissues (Okubara et al. 2014; Silva et al. 2012).

With the development of high-throughput techniques, omics data are providing opportunities for research into the molecular mechanisms of biological phenotypes (Kumar et al. 2015). From the perspective of systems biology, the occurrence and development of diseases are often a complicated process involving synergistic action among genes; yet, analyzing synergy among multiple genes is very difficult using traditional biological methods (Kim et al. 2009). The classic approach for identifying differentially expressed genes is to compare gene expression levels between experimental groups to produce a list of candidate genes that are differentially expressed according to a significance level (Childs et al. 2011). The development of this research approach focuses on the behavior of a single molecule, but it has been extended to the study of molecular interactions in dynamic network changes (Nutan et al. 2017). A co-expression network is a type of gene regulatory network, in which each node represents a gene and each edge two correlated genes based on their expression levels (Wang et al. 2014a, b). This network can reflect a set of gene expression correlations from a more systematic perspective, revealing how genes regulate each other and ultimately influence a phenotype (Garg et al. 2017). Studies have proven that correlation networks are useful for describing pairwise correlations between gene transcripts, and they are being increasingly employed in bioinformatics applications to explore the system-level functionality of genes (Zhang et al. 2018). Weighted gene co-expression network analysis (WGCNA), an approach that designates modules based on topological overlap, utilizes systems biology to explore associations between genes and aims to understand networks instead of individual genes (Langfelder and Horvath 2008). Furthermore, WGCNA has been shown to identify patterns that have been previously undetected in gene-to-gene comparison methods (Bao et al. 2017) and has become a common and useful strategy for investigating the causes of a disease or trait, as in a study of wheat resistance responses to powdery mildew (Zhang et al. 2016).

In contrast to the abovementioned advances, there has been little progress toward understanding the genetic networks involved in sheath blight resistance in rice at a transcriptomics scale. Several questions remain with regard to the pathogenesis of sheath blight, including (1) what are the systemic functions of cellular components after pathogen infection and (2) what are the differences in gene expression in resistant and susceptible host backgrounds. To address these questions, we used the standard TeQing rice genotype, which is moderately resistant, and a susceptible genotype, Lemont, as host plants to investigate *R. solani* AG1 IA infection. Based on our previous study (Zhang et al. 2017), we focused on comparing gene co-expression networks according to the transcriptional response of these two rice varieties to AG1 IA. The main objective of this study was to explore the genes and major modules involved in resistance against AG1 IA infection and to analyze relevant gene co-expression networks through WGCNA. Our research is expected to provide a rapid and efficient framework for constructing a more exact gene co-expression network with the aim of revealing gene expression changes after AG1 IA infection. The key components of this gene co-expression network represent promising candidates for devising effective strategies to control this destructive disease.

## Materials and methods

### Strains and rice varieties

The *R. solani* AG1 IA standard strain, which has been described previously (Yugander et al. 2014), was kindly provided by Prof. Er-Xun Zhou at the South China Agricultural University. Rice cultivars TeQing and Lemont are maintained in the Rice Research Institute of Sichuan Agricultural University.

### Comparison of transcriptional expression data

Based on our previous study, rice leaves inoculated with AG1 IA were maintained in a humidity chamber at 28 °C with a relative humidity greater than 80%. Leaves were harvested at 12, 24, 36, 48, and 72 h after AG1 IA infection, and 12 sets (contain control at 12 h) of RNA sequencing (RNA-Seq) data were generated in this study. Three biological replicates were created for each sample. For transcript profiling, leaf samples were collected, frozen in liquid nitrogen, and stored at − 70 °C. A total of 36 rice leaf samples, harvested at five different times after AG1 IA infection, were obtained for RNA-Seq, which was performed using the HiSeq PE125 by Biomarker Technologies. The detailed methods used for data processing and qRT-PCR validation and analysis are described in our previous article (Zhang et al. 2017). All of the transcriptome data are included in a Short Read Archive (SRA) (accession number SRP113646). Because these datasets were generated using material collected at different time points and from two rice varieties, expression levels in all samples were calculated uniformly from raw RNA-Seq data, as previously described (Wang et al. 2014a, b). The normalized data were used as inputs in our network inference program.

### Construction of weighted gene co-expression network and visualization

Co-expression networks were constructed using the WGCNA (v1.29) package in R (Langfelder and Horvath 2008); R language and Cytoscape were used for data visualization. The gene co-expression network is a scale-free weighted gene network. A significant feature of scale-free networks is that most nodes have only a few connections, with a few nodes having a large number of connections. To satisfy the precondition of scale-free network distribution, the adjacency matrix weight parameter β value needed to be determined. In this study, we evaluated β values from 1 to 20, and the corresponding correlation coefficient and mean value of the adjacent gene were calculated for each. A higher correlation coefficient (maximum = 1) indicates that the network is closer to the network size distribution. As a certain degree of gene connectivity, the β value should be as small as possible when the correlation coefficient is sufficiently large. For β less than 10, a larger β value indicates that the gene network is closer to being scale-free. Therefore, we selected β = 13 for TeQing and β = 11 for Lemont to construct the co-expression networks (Fig. S1). Based on the above analysis, we constructed a WGCNA to subdivide thousands of genes into several modules.

To describe the most common model of gene expression in each module, we conducted singular value decomposition of the gene expression values in every module and obtained multiple singular values and their corresponding eigenvectors. The characteristic vector with the highest degree of variation in gene expression in each module was defined as the characteristic gene expression of the module. We extracted the featured genes of each module for the 36 rice leaf samples and then calculated their association with the two genotypes (TeQing and Lemont). We drew heat maps for each module based on correlation coefficients, with a deeper color representing a higher degree of correlation. To further explore interactions among genes in each module, we selected those genes with the highest connectivity to draw the gene network. In addition, information regarding the functions of differentially expressed genes was collected from unigene annotations, and these genes were subjected to Gene Ontology (GO) and Kyoto Encyclopedia of Genes and Genomes (KEGG) significant enrichment analyses to identify the biological functions and metabolic pathways in which these genes participate.

### Co-expression network construction based on hub genes

For network biology analysis, hub genes are good representatives of each module relative to other genes in the module and have important biological significance in system analysis (Sriroopreddy and Sudandiradoss 2018). For comparing the difference in resistance between TeQing and Lamont, we selected early and later modules with high correlations to assess the mechanisms of disease resistance. We chose representative hub genes with a high degree of connection in the early and later modules to analyze differences between the two rice varieties. For Lemont, we chose the early turquoise module and the later brown module (Fig. 4). For TeQing, we selected the early yellow module and the later Black module (Fig. 5). To further investigate interaction among genes within each module, we selected the first 50 genes with the highest connectivity within the module to map the gene network.

To further compare differences in disease resistance between TeQing and Lemont, we considered the top 500 genes with the highest connectivity across the interaction networks of both varieties as core genes. Additionally, we searched for disease resistance genes in Oryzabase (https://shigen.nig.ac.jp/rice/oryzabase/) and obtained 3601 genes, among which 1505 appear in our data (Table S4; Table S5). We analyzed these 1505 resistance genes compared with the 500 core genes and extracted core resistance genes to map the co-expression network. TeQing and Lemont network maps were drawn separately.

## Results

### Global gene inference for gene co-expression analyses

Based on pairwise correlations between genes in common expression trends across all samples, 11,947 candidate regulatory genes were identified between TeQing and Lemont; the average transcripts per million (TPM) were higher than 10 for all 36 samples. We established the rice gene co-expression network using WGCNA, and a global gene expression data matrix was generated based on combined and normalized expression data for TeQing and Lemont. The co-expression network was constructed using the 11,947 selected genes, and clusters of highly co-expressed genes were detected and assigned to module colors based on a previously reported method (Medina and Lubovac-pilav 2016; Guo et al. 2016). Because the expression data reflect different biological processes at different time points with differential expression after AG1 IA infection, we aimed to construct a reduced network structure that describes the regulation of fundamental biological processes with an emphasis on control of rice resistance to AG1 IA. This network can be further fitted with expression data from different time points to understand the regulation of specific biological functions.

### Co-expression network analysis by WGCNA

**Fig. 1:**
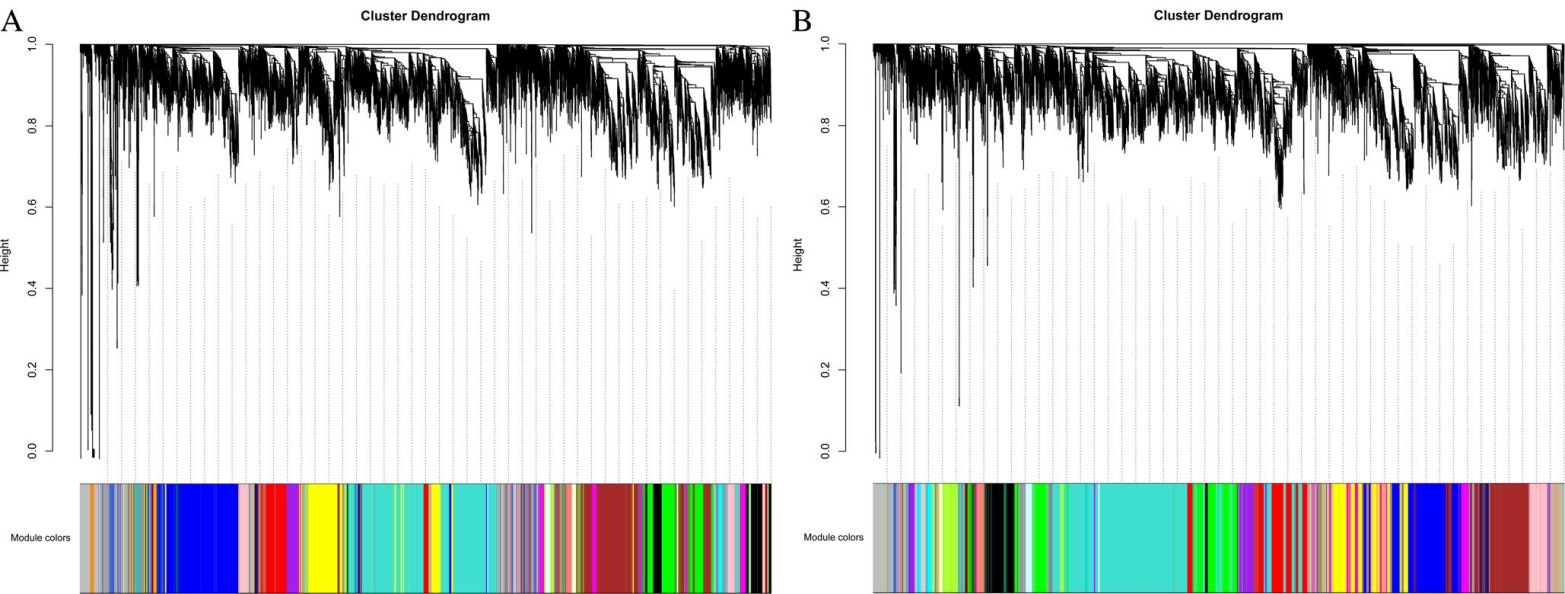
WGCNA of genes in leaf tissues of TeQing (**a**) and Lemont (**b**) after AG1 IA infection. Hierarchical cluster trees show the co-expression modules identified by WGCNA

**Fig. 2:**
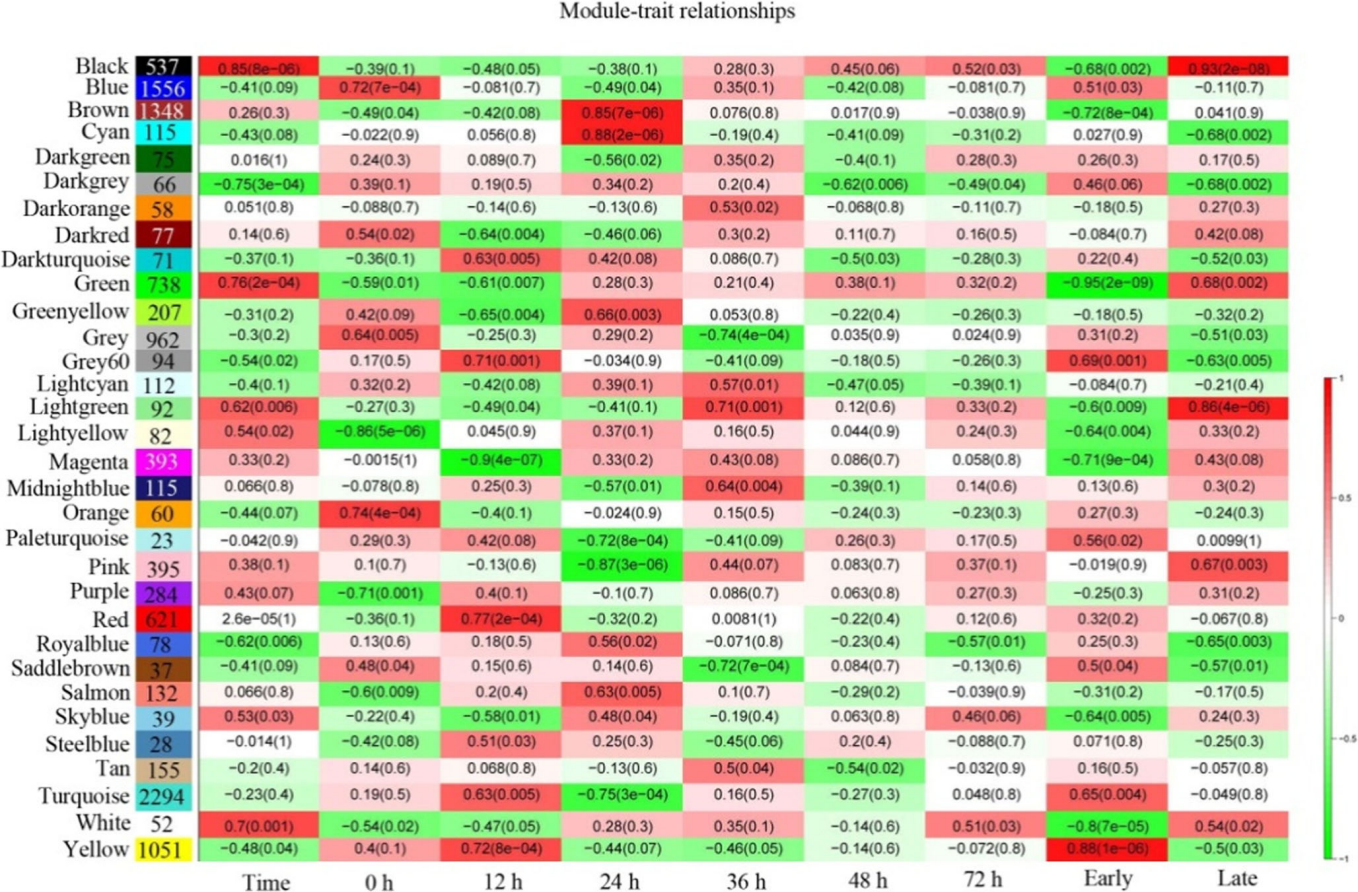
Matrix showing Module-Trait Relationships (MTRs) for TeQing. Each row corresponds to a module. The number of genes in each module is indicated on the left. Each column corresponds to a time result. The MTRs are colored based on their correlation: red indicates a strong positive correlation and green indicates a strong negative correlation

**Fig. 3:**
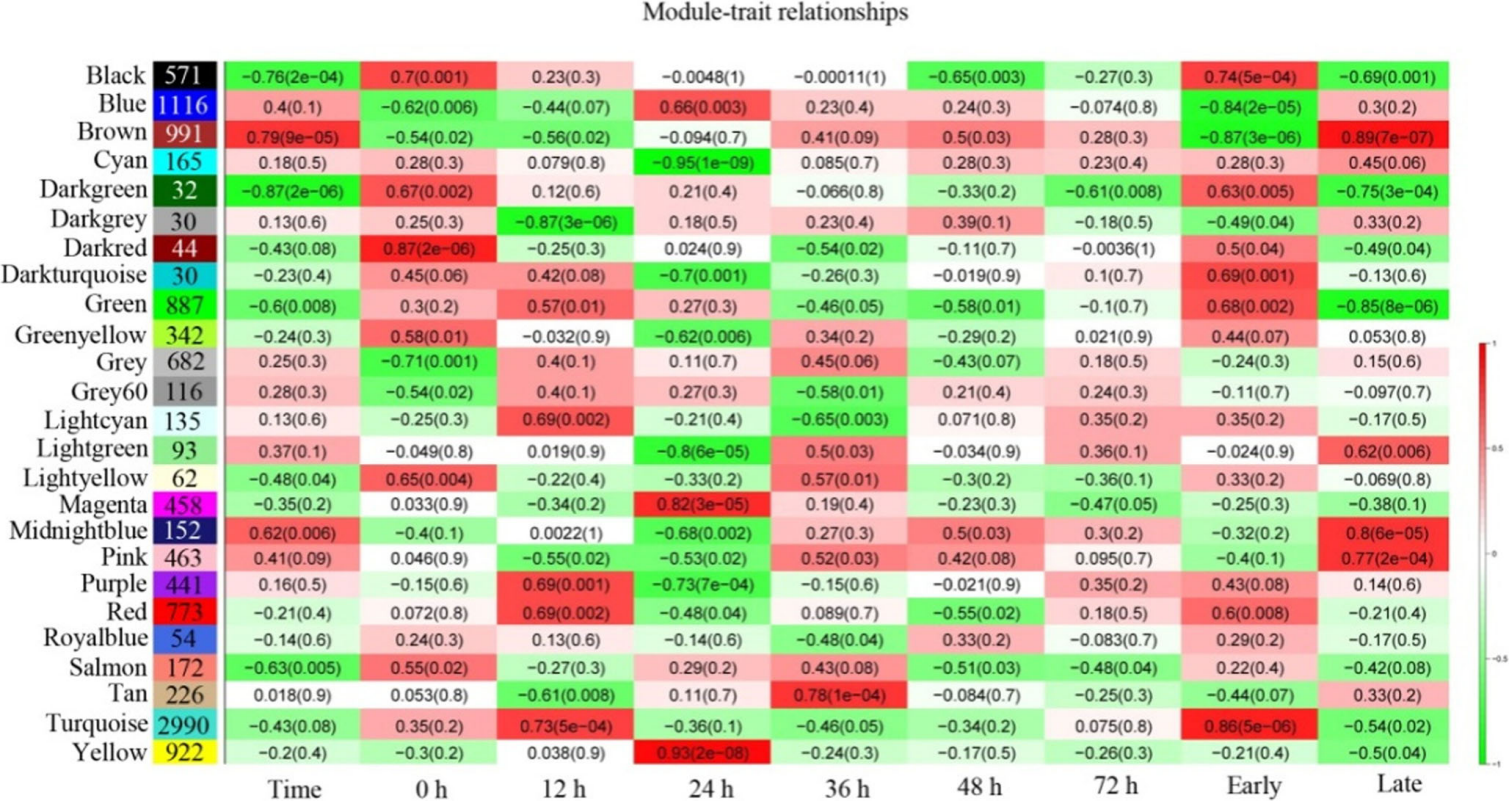
Matrix showing Module-Trait Relationships (MTRs) for Lemont. Each row corresponds to a module. The number of genes in each module is indicated on the left. Each column corresponds to a time result. The MTRs are colored based on their correlation: red indicates a strong positive correlation and green indicates a strong negative correlation

We performed WGCNA for each rice cultivar separately. Each tree branch constitutes a module, and each leaf in the branch represents one gene, as shown in the hierarchical clustering tree (Fig. 1). For further analysis, we cut the tree from the resulting dendrogram into isolate modules (clusters). Sets of genes (modules) with common expression patterns were identified based on their correlations with time. WGCNA resulted in 32 and 26 distinct modules for TeQing and Lemont, respectively, as shown by the dendrogram. The number of target genes for each module ranged from 23 to 2294 for TeQing (Fig. 2) and from 30 to 2990 for Lemont (Fig. 3). The matrix representing all Module-Trait Relationships (MTRs) is distinct. These modules contain genes that are either positively or negatively correlated and that have expression levels that are either high or low after AG1 IA infection. The results show that some modules are highly correlated with AG1 IA infection. In addition, the modules were selected for further examination at MTRs > 0.7, and genes were retained in each module for further analysis based on their intra-module connectivity.

### Modules associated with differences between TeQing and Lemont after AG1 IA infection

As we found in our preliminary study (unpublished data), 24-h post-AG1 IA infection was an important time. Thus, we added two data processing analyses of early (before 24 h) and later (after 24 h) time points during infection (Figs. 2 and 3). The identification of rice genotype-specific modules after AG1 IA infection was of particular interest. We identified modules significantly associated with time for both TeQing and Lemont. For TeQing, the Black module was highly correlated with resistance throughout the experimental period (*r* = 0.85, *p* = 8e-06), especially in the later stage (*r* = 0.93, *p* = 2e-08). The red module (*r* = 0.77, *p* = 2e-04) at 12 h, the brown (r = 0.85, *p* = 7e-06) and cyan modules (*r* = 0.88, *p* = 2e-06) at 24 h and the light green module at 36 h showed high correlation with AG1 IA infection (Fig. 2). In addition, for Lemont, turquoise (*r* = 0.86, *p* = 5e-06) at 12 h, yellow (*r* = 0.93, *p* = 2e-08) and magenta (*r* = 0.82, *p* = 3e-05) at 24 h and tan (*r* = 0.78, *p* = 1e-04) at 36 h were highly correlated with AG1 IA infection. In the later stage (after 24 h), the midnight blue (*r* = 0.8, *p* = 6e-05), pink (r = 0.77, *p* = 2e-04) and brown (*r* = 0.89, *p* = 7e-07) modules showed high correlation, with the brown module (*r* = 0.79, *p* = 9e-05) having a high correlation over the entire experimental period (Fig. 3).

To identify features of each module that indicate their biological roles in response to AG1 IA infection, functional annotations of the disease-related modules were performed based on their gene compositions (*p* < 0.05). For TeQing, according to gene functional annotations, the brown module was significantly enriched in genes involved in phenylalanine, tyrosine and tryptophan biosynthesis, biosynthesis of amino acids, carbon metabolism, plant-pathogen interaction, and alpha-linolenic acid metabolism (Table S1). Moreover, other resistance-related secondary metabolic pathways, such as flavone and flavonol biosynthesis/flavonoid biosynthesis, phenylalanine metabolism/phenylpropanoid biosynthesis, and sphingolipid metabolism, also exhibited significant enrichment. The genes in the cyan and yellow modules are mainly involved in photosynthesis. In addition, plant hormone signal transduction and terpenoid backbone biosynthesis were significantly enriched in the yellow module. For turquoise, which was the largest module, gene enrichment in photosynthesis and other metabolic pathways was also found.

For Lemont, the turquoise module displayed significant enrichment in photosynthesis, photosynthesis-antenna proteins and porphyrin and chlorophyll metabolism (Table S2). We also found photosynthesis and nutrient material metabolism to be significantly enriched based on GO terms. For yellow and magenta, the most highly enriched genes at 24 h were found to be related to secondary metabolites in plant resistance; for example, phenylalanine, tyrosine and tryptophan biosynthesis, phenylalanine metabolism/phenylpropanoid biosynthesis, riboflavin metabolism, flavonoid biosynthesis and stilbenoid, diarylheptanoid and gingerol biosynthesis. The products of these pathways have been shown to possess antioxidant and antimicrobial properties. In the brown module, diterpenoid biosynthesis, alpha-linolenic acid metabolism, oxidative phosphorylation and valine, leucine and isoleucine degradation were significantly enriched. Ascorbate and aldarate metabolism and peroxidation were also enriched. Based on the variation in enrichment among the above modules, especially comparing resistance-related modules between early and later stages, the resistance process appears to mainly involve the metabolism of some resistance-related substances, along with a burst of reactive oxygen species (ROS) at the early stages of infection. Over a prolonged duration of infection and with the spread of the disease, relevant defense response modules appear to interact to participate in resistance responses.

### Hub gene selection for TeQing and Lemont co-expression networks

**Fig. 4:**
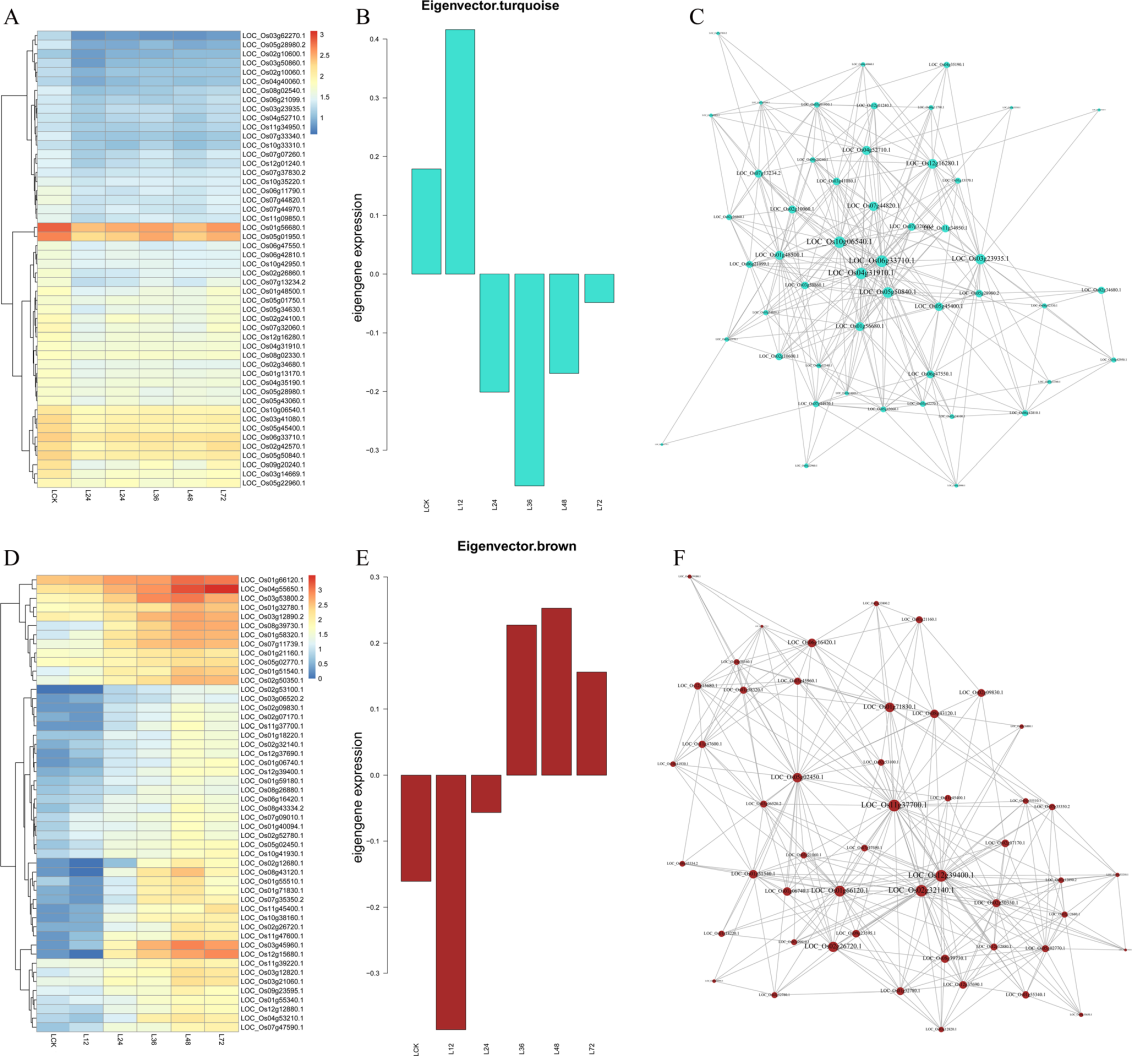
Co-expression network analysis of turquoise and brown modules in Lemont. **a**, **d** Heatmaps showing the genes that were significantly over-represented in the turquoise and brown modules, respectively. **b**, **e** Eigen-gene expression profiles for the turquoise and brown modules at different times. The *y*-axis indicates the value of the module Eigen-gene and the *x*-axis indicates the time of sample collection. **c**, **f** The correlation networks corresponding to the turquoise and brown modules, respectively. Candidate hub genes are shown as filled circles

As indicated by heatmaps (Fig. 4a), turquoise-module-specific genes were over-represented in the early stage in Lemont (Table S3). Eigen-gene expression profiles for the turquoise module are shown in Fig. 4b, with 27 hub genes in the turquoise module encoding expressed proteins. Four genes encode transcription factors (TFs), including MYB family TFs, OsbZIP14, and two TF-like proteins. Other genes, such as glycosyltransferases, OsGT1, and serine hydroxymethyltransferase 1 (OsSHM1), were also found. The correlation network of the turquoise module is shown in Fig. 4c. The membrane-trafficking protein OsVAMP714, oxidoreductase, heavy-metal ATPase OsHMA1, ubiquitin-conjugating enzyme E2, ferredoxin-thioredoxin reductase, imidazoleglycerol-phosphate dehydratase, and spermidine synthase were identified as candidate hub genes for this module.

Brown module-specific gene heatmaps (Fig. 4d) were over-represented in the later stage in Lemont (Table S3). Eigen-gene expression profiles for the brown module are shown in Fig. 4e, with 12 genes encoding expressed proteins, all of which are TFs, including three MYB family TFs, two WRKY TFs, three bZIP TFs, three NAC TFs, and a Dof TF. The correlation network of the brown module is shown in Fig. 4f. Genes encoding cytidine/deoxycytidylate deaminase, allene oxide synthase OsAOS3, OsNAC6, endo-β-1,3-glucanase OsGLN1, glycine-rich cell wall structural protein precursor, plant PDR ABC transporter-associated domain-containing protein, inositol 1,3,4-trisphosphate 5/6-kinase-like gene, and AP2 domain-containing protein were identified as candidate hub genes for this module.

**Fig. 5:**
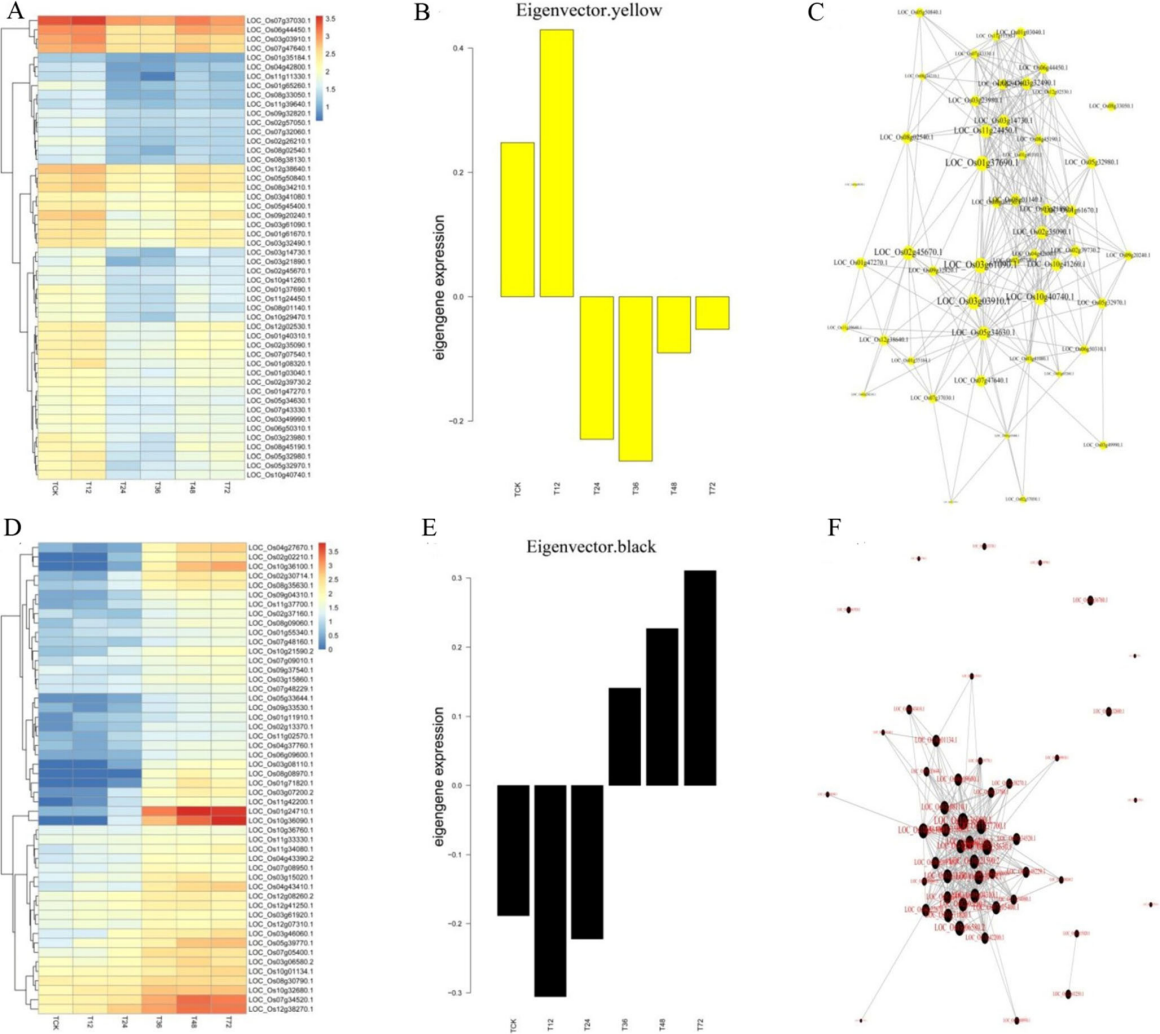
Co-expression network analysis of yellow and black modules in TeQing. **a**, **d** Heatmaps showing the genes that were significantly over-represented in the yellow and black modules, respectively. **b**, **e** Eigen-gene expression profiles for the yellow and black modules at different times. The *y*-axis indicates the value of the module Eigen-gene and the *x*-axis indicates the time of sample collection. **c**, **f** The correlation networks corresponding to the yellow and black modules, respectively. Candidate hub genes are shown as filled circles

For TeQing, heatmaps (Fig. 5a) showed over-representation in the early stage of yellow-module-specific genes (Table S3). The Eigen-gene expression profiles for the yellow module are shown in Fig. 5b. In total, 18 genes encode expressed proteins: six encode TFs, including a Dof TF, MYB-like DNA-binding domain-containing protein, a GRAS family TF, a bZIP TF, and two MYB family TFs. Other genes, such as OsHAK8, proton gradient regulation 5, aldehyde dehydrogenase, and zinc finger, C3HC4-type domain-containing protein, were also found. OsCATC, OsCAX1a, OsIAA1, DUF1230 domain-containing protein, and adenylate kinase were identified as candidate hub genes for this module (Fig. 5c).

According to the heatmaps for TeQing in the later stage (Fig. 5d), Black-module-specific genes are over-represented (Table S3). The Eigen-gene expression profiles for the Black module are shown in Fig. 5e, with eight of 53 genes encoding expressed proteins. One gene encodes the Dof TF OsDof-6. The correlation network of the Black module is shown in Fig. 5f. MTN26L2-MtN26 family protein precursor, germin-like protein, and serine carboxypeptidases were identified by WGCNA as candidate hub genes for this module. These results indicate a markedly higher degree of infection in Lemont versus TeQing. Our results show that both hub gene networks have a large number of expressed protein genes with unknown functions; further study is warranted, as these proteins may have important functions.

**Fig. 6:**
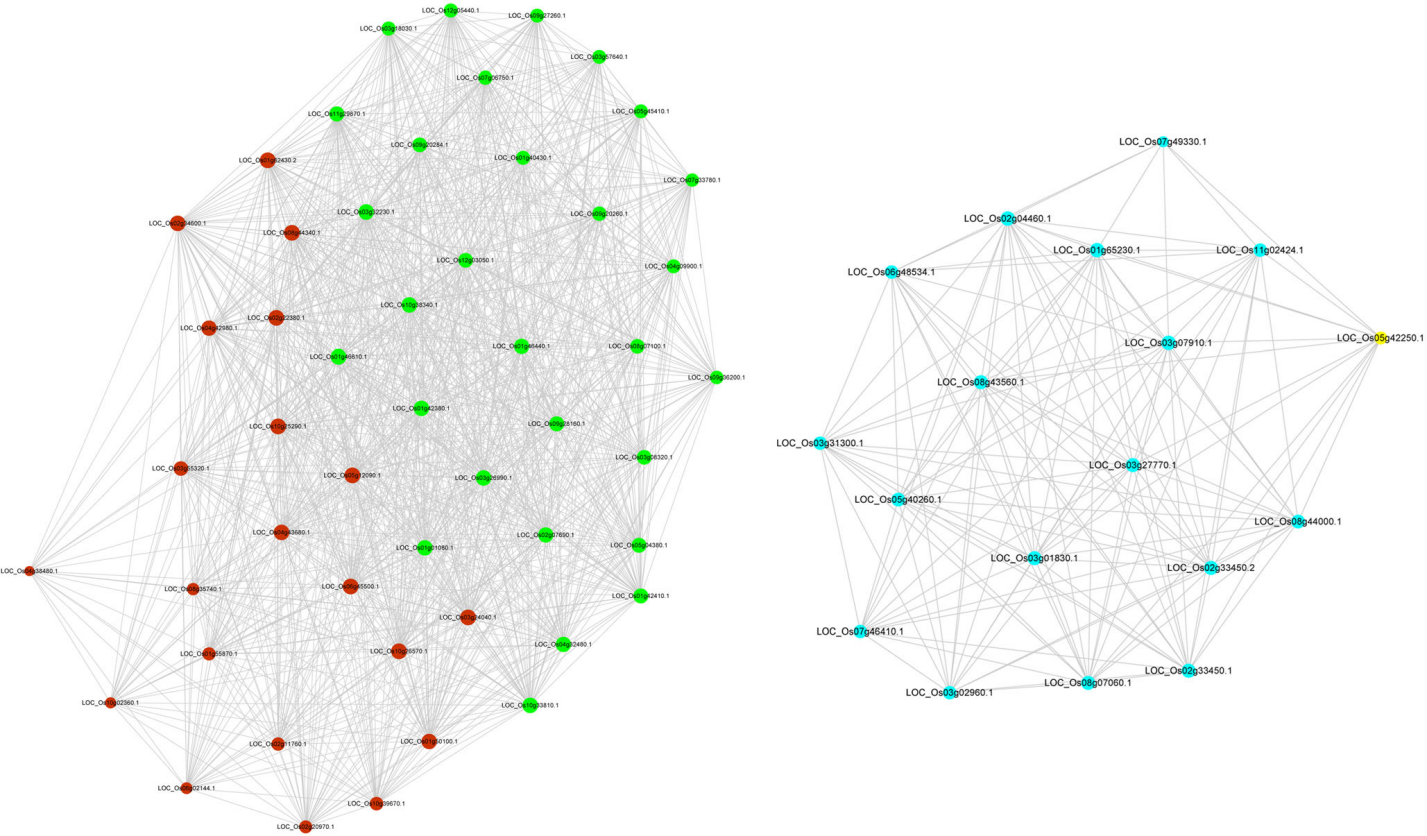
Core disease resistance gene network for TeQing derived from comparing the core genes with known resistance genes

**Fig. 7:**
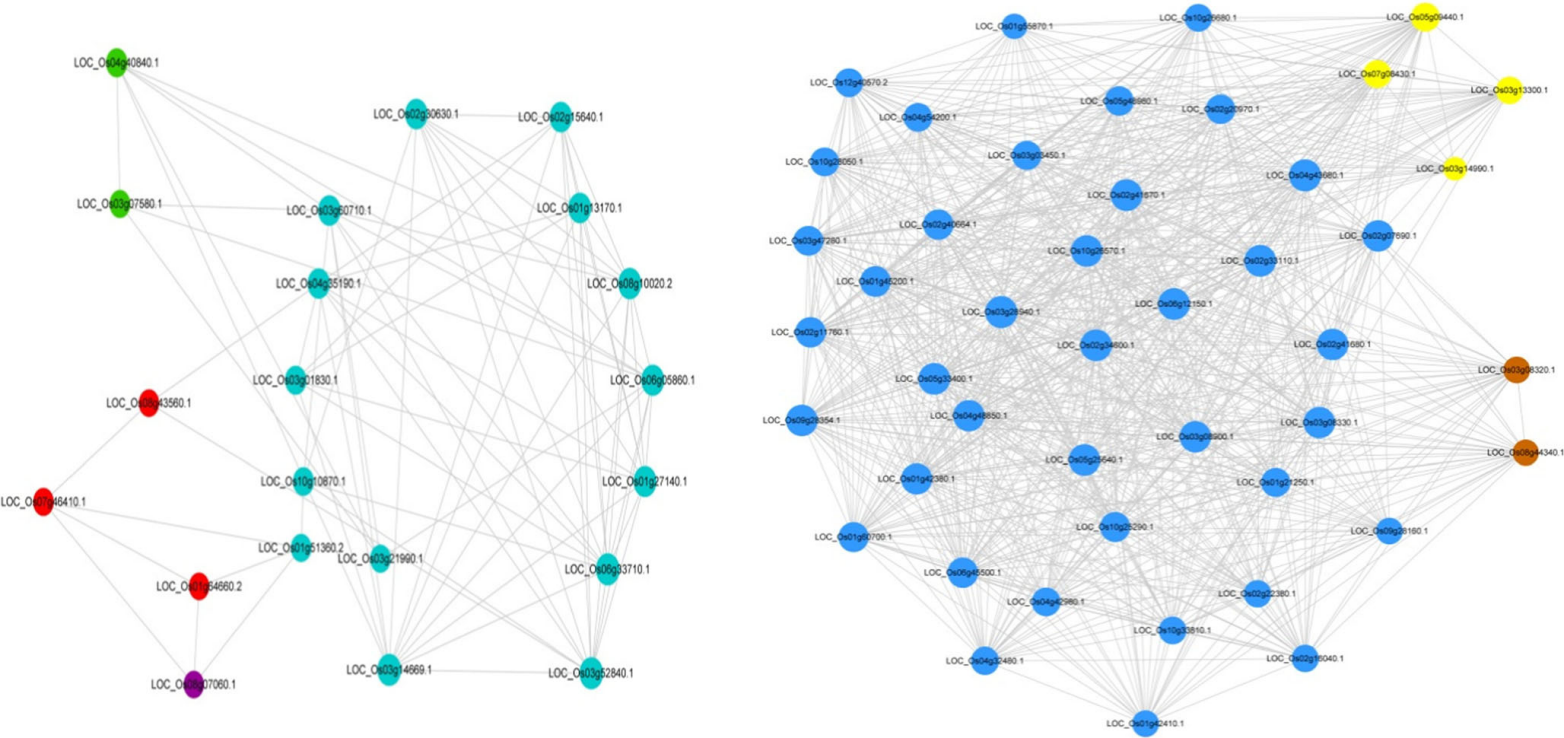
Core disease resistance gene network for Lemont derived from comparing the core genes with known resistance genes

The analysis revealed the core disease resistance gene network operating in rice sheath blight resistance, and the results showed a common core of resistance genes in multiple networks. Disease resistance core genes were divided into two major blocks according to the degree of connection between co-expressed genes, and the aggregation of co-expression of core resistance genes reflected two major gene expression patterns. In TeQing, the core disease resistance genes were mainly distributed into three modules: dark red, turquoise, and green. A network of representative genes was drawn, and the results are provided in Fig. 6. Genes associated with a high degree of connection included glutathione S-transferase, ascorbate peroxidase, VQ motif-containing protein, jasmonate ZIM-domain proteins (OsJAZ12, OsJAZ11, and OsJAZ5), and some TFs (WRKY, MYB, and NAC). For Lemont, the core disease resistance genes were mainly distributed in two modules: turquoise and blue (Fig. 7). These modules include OsPAL3, phosphofructokinase, jasmonate ZIM-domain proteins (OsJAZ6, OsJAZ11, OsJAZ10, OsJAZ12, and OsJAZ5), VQ motif-containing protein, shikimate kinase, ubiquitin-conjugating enzyme, and some TFs (WRKY, MYB, and NAC). Overall, the two genotypes share some common aspects for sheath blight resistance. However, differences in the specific regulatory gene networks involved were noted.

## Discussion

Given that expression of a large group of genes was affected by AG1 IA infection, we used WGCNA to construct a gene co-expression network to identify differences between modules. The main purpose was to search the rice sheath blight response mechanism and reveal the key genes and major modules involved in the responses of two types of rice with different resistance levels to AG1 IA. Based on WGCNA and comparison of gene co-expression networks, we provide the first systems view of the rice response to AG1 IA infection at the transcriptome level. To our knowledge, no report about gene-gene interaction networks of rice sheath blight is available to date. The signal transduction pathways and defense processes involved in plant disease resistance are highly complex and relate to multiple genes. Previous studies have shown that network analysis is a very effective method for uncovering genes and interactions that functionally relate to plant disease resistance (Lee et al. 2011; Mukhtar et al. 2011; Weßling et al. 2014). Interestingly, this network includes pathways that are important for plant defense against necrotrophic fungi at different time points after infection, such as oxide-reduction, stress response, and other biosynthetic processes. Therefore, this work facilitates identification of the hub gene sets and major modules associated with disease resistance.

Several modules were selected for further analysis and discussion, as explained above. To examine the relevance of the distribution of these genes and their biological roles in the two genotypes, the biological significance of the genes was evaluated on the basis of their composition using GO and KEGG analyses. The functions of genes that have known biological functions can be predicted based on their module, and this analysis identified a broad range of biological processes that were affected by AG1 IA infection. Both primary and secondary genes were significantly affected by the pathogen during the processes associated with resistance metabolism. In the early (12 h) stage in both TeQing and Lemont, we found photosynthesis to be the common element of the metabolic pathways involved in resistance. This pathway was also more enriched in TeQing than in Lamont at the early stage. In addition, photosynthesis was shown to be crucial in the early stages of the overall resistance process. Some studies have noted a reduction in photosynthesis after pathogen infection and that normal photosynthesis is disrupted by photosynthetic organ damage (Pérez-Clemente et al. 2015). At the same time, photosynthesis provides the material for other resistance-related metabolites (Major et al. 2010). Comparing the available metabolic materials of the two varieties, 24 h was found to be an important time point for pathogen invasion. Indeed, a number of gene functions involving resistance-related metabolites showed significant enrichment during this period, such as Flavone and flavonol biosynthesis/flavonoid biosynthesis and Phenylalanine metabolism/phenylpropanoid biosynthesis. Previous studies have found that phenylalanine metabolism is closely related to plant disease resistance and is therefore an important index used to measure plant disease resistance (Fu et al. 2015). The branches downstream of phenylalanine metabolism mainly include the lignin synthesis and flavonoid synthesis pathways, which produce phenylpropanoid derivatives such as coumarin, flavonol, and lignin. These phenylpropanoid metabolites act as plant antitoxins, cell wall structural elements, and signal transduction molecules and play important roles in plant disease resistance (Ganapathy et al. 2016; Lozoya-Saldana et al. 2007; Jubault et al. 2013). In addition, the metabolism of alpha-linolenic acid, which is associated with the processing of jasmonic acid (JA), showed a significant accumulation in TeQing at 24 h. This finding suggests that primary metabolism is involved in the resistance process at the beginning of AG1 IA infection and that JA plays an important role at 24 h and thereafter. With the further spread of the pathogen in the leaf, the related disease resistance processes appear to form a complex, three-dimensional resistance network. Overall, the gene expression analyses revealed notable alterations in the transcriptional levels of genes related to plant metabolism, suggesting some roles of primary host metabolism in relation to defense mechanisms.

We employed WGCNA to construct gene networks representing early and later infection stages with a high degree of connection in TeQing and Lemont. Comparing the hub genes of the two varieties, the resistance gene network of TeQing was more populous than that of Lemont in the early stage, whereas Lemont was significantly enriched at 24 h, with higher connectivity among its related genes. In TeQing, OsIAA1 and SLR1 had a high degree of connectivity in the network. Previous studies have shown that auxin plays an important role in the hormone-signaling network involved in the regulation of defense responses against some necrotrophic pathogens (Naseem et al. 2015). In addition, changes in auxin can be due to the indirect effects of the JA and ethylene (ET) pathways, because these hormones affect the signaling, transport, and biosynthesis of auxin (Saini et al. 2013) or are a direct effect of a pathogen on the auxin pathway (Mah et al. 2012). However, further confirmation of the critical role of the level of auxin for affecting the balance of other hormones and in fine-tuning defense responses specific to AG1 IA remains to be discovered. Studies have found that SLR1 can regulate plant resistance by integrating and enhancing salicylic acid and JA signals (Vleesschauwer et al. 2016). ROS control has many different processes and has an important function in plant disease resistance (Mittler et al. 2004). Active oxygen-related genes such as OsCATC and chloroplast glutathione peroxidase were found in the hub gene network, and by encoding important enzymes scavenging ROS in plants, these genes play a role in the elimination of ROS to maintain normal ROS levels in plants (Lin et al. 2012; Chang et al. 2009). In Lemont, some of the genes associated with stress resistance showed higher connectivity during the early stage of infection, such as OsVAMP714; previous research has shown that OsVAMP714 plays an important role in rice blast resistance as well as in vegetative growth (Sugano et al. 2016). In the later stage, allene oxide synthase was associated with JA synthesis at a high degree of connectivity, demonstrating that JA has a crucial function in sheath blight resistance. Other disease-related genes and expressed proteins were identified in the network, and their roles in sheath blight resistance must be further verified.

Transcriptional regulation plays an important role in modulating gene expression and transmitting stress signals in plants (Chen and Zhu 2004). Because they control many downstream genes, TFs are potential tools for manipulating stress tolerance, and both gene-for-gene resistance and basal disease resistance are mediated by a series of TFs, including MYB, NAC, WRKY, and basic region/leucine zipper (bZIP) family members (Kim et al. 2015; Pandey and Somssich 2009; Singh et al. 2002). Based on these results, a considerable number of TFs among hub genes are involved in resistance-related processes, such as Dof TFs, which are involved in many plant metabolic processes (Shaw et al. 2009). In Arabidopsis, Dofs are involved in phenylalanine and flavonoid synthesis pathways. For example, AtDOF4;2 was identified as being potentially involved in the regulation of phenylpropanoid metabolism in Arabidopsis (Skirycz et al. 2007). WRKY TFs are crucial regulatory components of plant responses to pathogen infection (Gallou et al. 2012). Previous studies have reported that JA plays an important role in WRKY30-mediated defense responses to *R. solani* and *Magnaporthe grisea*, and WRKY30 improves sheath blight resistance in rice by regulating expression of relevant resistance genes (Peng et al. 2012). MYB TFs are considered to be involved in primary and secondary metabolism (such as phenylpropanoid metabolism), hormone signal transduction, and responses to biotic and abiotic stresses (Liu et al. 2015; Zhao et al. 2013). TFs within the network and their cross-linked pathways are mostly involved in signal transduction, oxide-reduction processes, and defense responses. Understanding the mechanisms of TFs involved in resistance processes will not only be helpful for elucidating the plant TF-mediated signal transduction network controlling sheath blight resistance but will also provide a theoretical basis for the discovery and application of new resistance-related genes.

Core resistance genes are important markers in the process of plant disease resistance. We identified an interesting phenomenon in this study: several VQ proteins had high connectivity to core resistance gene networks in TeQing and Lemont. VQ proteins are plant-specific proteins and comprise a multigene family in a wide variety of plant species (Jing and Lin 2015). Expression of VQ genes is induced or suppressed by salicylic acid (SA), JA, or pathogen treatment, suggesting that these genes may have a key role in the plant response to disease (Li et al. 2014). Xie et al. (2010) found that overexpression of SIB1 (a VQ motif-containing protein) causes plants to activate defense-related genes following pathogen infection or SA and JA treatments, leading to enhanced resistance to *P. syringae* infection. Moreover, Wang et al. (2015a, b) found VQ12 and VQ29 to be highly responsive to *B. cinerea* infection, and VQ12 and VQ29 might be partially involved in the JA-signaling pathway, as demonstrated via expression analysis of defense-signaling mutants. In the present study, jasmonate ZIM-domain proteins (JAZs) were found in the core gene network. JA is an important signaling molecule in resistance to necrotrophic pathogens (Ranjan et al. 2015). JAZs are repressor proteins that participate in many signaling pathways, particularly as JA pathway inhibitors, and play a key role in regulating the host immune process (Major et al. 2017). JAZs respond to JA stimulation to release MYC2, which then initiates the transcription of JA-responsive genes (Sasaki-sekimoto et al. 2013). Furthermore, JAZs play an important regulatory role as hub proteins in the process of plant-hormone-mediated signal transduction (Wasternack and Hause 2013).

## Conclusions

In summary, this study compared various networks to shed new light on genes regulated through two types of rice responses to AG1 IA at the transcriptome scale, providing a reliable basis for further investigation into the important mechanisms and critical genes that are likely crucial for rice responses to AG1 IA. This initial gene co-expression analysis based on WGCNA provides a regulatory framework that links every gene at the transcriptome level. Time point association analysis showed that several of the identified top target genes might be critical for disease resistance. Although the distinctions between the modules are clear, we found that some genes are commonly associated with more than one module, which reflects the complexity of the regulatory networks. Overall, analysis of these modules offers valuable information on the gene regulatory pathway that controls pivotal biological processes in disease responses. Furthermore, future research involving the functional characterization of these regulators and target genes through experimental approaches will allow a better understanding of rice-*R. solani* interactions at the systems biology level. The gene co-expression networks will be very useful for researchers seeking to visualize the sub-networks specific to certain biological processes or searching for potential gene-gene interactions for individual genes or groups of genes.

## Electronic supplementary material

Fig. S1 (JPEG 2351 kb)

Table S1 (DOCX 26 kb)

Table S2 (DOCX 24 kb)

Table S3 (XLSX 96 kb)

Table S4 (XLSX 351 kb)

Table S5 (XLSX 350 kb)
