# Supplementary figures and images for: Comparison of gene co-networks reveals the molecular mechanisms of the rice (Oryza sativa L.) response to Rhizoctonia solani AG1 IA infection
Source: Funct Integr Genomics. 2018 May 5;18(5):545–57. doi: 10.1007/s10142-018-0607-y (PMC6097106; doi:10.1007/s10142-018-0607-y)

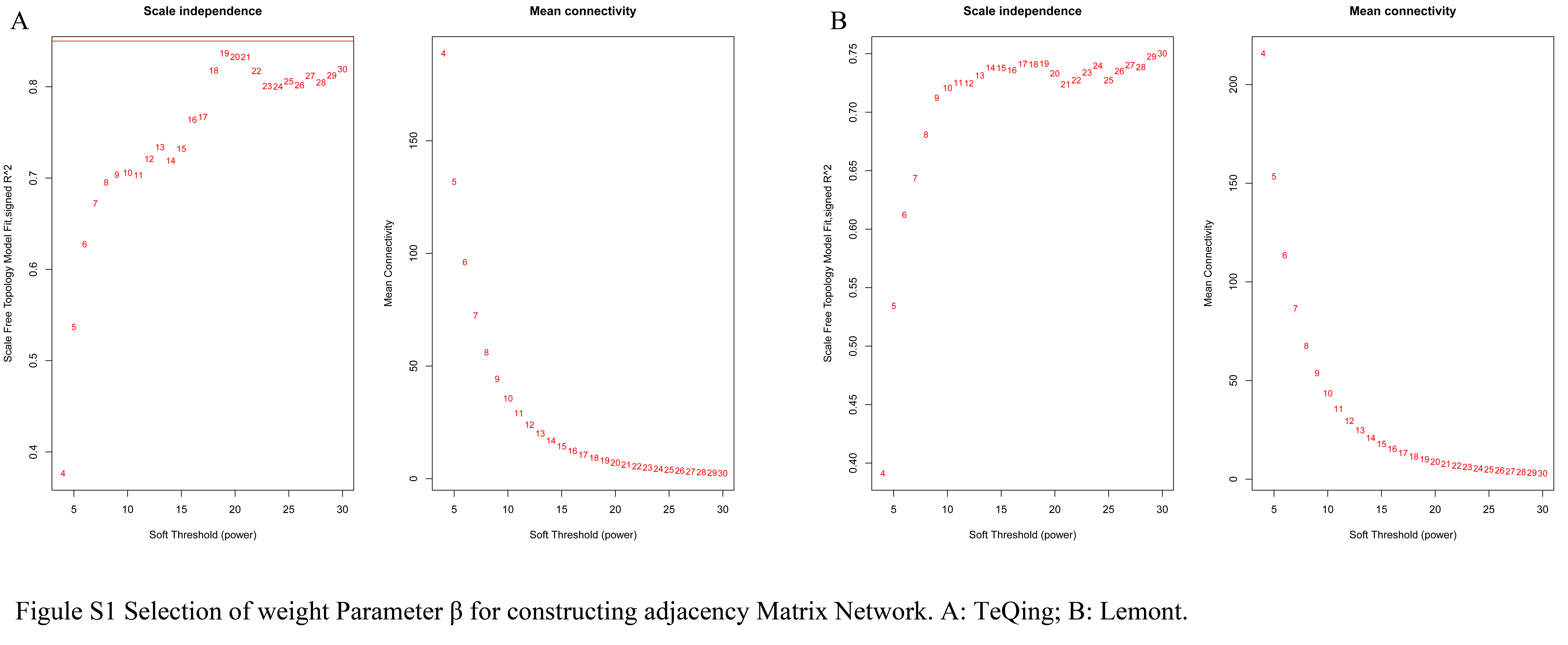

Supplement: Supplementary file 1 — (JPEG 2351 kb) [file 10142_2018_607_MOESM1_ESM.jpg]
