# Supplementary material for: Comparison of gene co-networks reveals the molecular mechanisms of the rice (Oryza sativa L.) response to Rhizoctonia solani AG1 IA infection
Source: Funct Integr Genomics. 2018 May 5;18(5):545–57. doi: 10.1007/s10142-018-0607-y (PMC6097106; doi:10.1007/s10142-018-0607-y)
Supplement: Supplementary file 2 — (DOCX 26 kb) [file 10142_2018_607_MOESM2_ESM.docx]

| Table S1. Overview of the significantly overrepresented KEGG pathways and GO terms associated with the modules detected using WGCNA in TeQing. | | | | | |
| --- | --- | --- | --- | --- | --- |
| WGCNA module | KEGG pathway or GO-term | pvalue | p.adjust | gene Count | Enrich.score |
| Brown | KEGG: Phenylalanine, tyrosine and tryptophan biosynthesis | 4.36E-14 | 4.01E-12 | 24 | 6.305545 |
|  | KEGG: Biosynthesis of amino acids | 2.22E-11 | 1.02E-09 | 54 | 2.610159 |
|  | KEGG: Citrate cycle (TCA cycle) | 0.000109 | 0.00334 | 16 | 2.864057 |
|  | KEGG: Carbon metabolism | 0.000245 | 0.004658 | 42 | 1.736426 |
|  | KEGG: Amino sugar and nucleotide sugar metabolism | 0.000253 | 0.004658 | 23 | 2.20385 |
|  | KEGG: Plant-pathogen interaction | 0.000389 | 0.00596 | 22 | 2.185153 |
|  | KEGG: alpha-Linolenic acid metabolism | 0.000571 | 0.007506 | 11 | 3.143554 |
|  | KEGG: Protein processing in endoplasmic reticulum | 0.000897 | 0.01032 | 33 | 1.762452 |
|  | KEGG: Endocytosis | 0.001237 | 0.012646 | 21 | 2.048358 |
|  | KEGG: Protein export | 0.001476 | 0.013577 | 13 | 2.55134 |
|  | KEGG: Phenylpropanoid biosynthesis | 0.003562 | 0.029794 | 20 | 1.916391 |
|  | KEGG: N-Glycan biosynthesis | 0.004093 | 0.031378 | 10 | 2.627311 |
|  | KEGG: Sphingolipid metabolism | 0.004666 | 0.03302 | 7 | 3.257865 |
|  | KEGG: Glycolysis / Gluconeogenesis | 0.010242 | 0.061591 | 22 | 1.690402 |
|  | KEGG: Flavone and flavonol biosynthesis | 0.010817 | 0.061591 | 2 | 10.85955 |
|  | KEGG: Phagosome | 0.011065 | 0.061591 | 14 | 1.965953 |
|  | KEGG: Flavonoid biosynthesis | 0.011381 | 0.061591 | 5 | 3.541158 |
|  | KEGG: Propanoate metabolism | 0.018306 | 0.092654 | 6 | 2.792456 |
|  | KEGG: Selenocompound metabolism | 0.019135 | 0.092654 | 5 | 3.132563 |
|  | KEGG: Phenylalanine metabolism | 0.023226 | 0.106838 | 13 | 1.841402 |
|  | KEGG: Stilbenoid, diarylheptanoid and gingerol biosynthesis | 0.036404 | 0.159483 | 4 | 3.102729 |
|  | KEGG: Pyruvate metabolism | 0.042821 | 0.179069 | 14 | 1.65254 |
|  | GO: Golgi apparatus | 1.44E-19 | 1.15E-18 | 62 | 2.381335 |
|  | GO: peroxisome | 0.001124 | 0.004497 | 22 | 1.437012 |
|  | GO: endosome | 0.039021 | 0.104055 | 10 | 1.46996 |
| Turquoise | KEGG: Glyoxylate and dicarboxylate metabolism | 0.001419 | 0.078196 | 17 | 2.213309 |
|  | KEGG: Glycine, serine and threonine metabolism | 0.001533 | 0.078196 | 16 | 2.262307 |
|  | KEGG: Aminoacyl-tRNA biosynthesis | 0.004987 | 0.122522 | 15 | 2.076262 |
|  | KEGG: Porphyrin and chlorophyll metabolism | 0.005606 | 0.122522 | 11 | 2.37125 |
|  | KEGG: Ribosome | 0.006006 | 0.122522 | 42 | 1.4767 |
|  | KEGG: Photosynthesis | 0.008116 | 0.137966 | 11 | 2.260098 |
|  | KEGG: One carbon pool by folate | 0.009532 | 0.138898 | 6 | 3.155918 |
|  | KEGG: Carbon fixation in photosynthetic organisms | 0.01517 | 0.193416 | 17 | 1.760191 |
|  | KEGG: Tropane, piperidine and pyridine alkaloid biosynthesis | 0.026638 | 0.272729 | 5 | 2.858622 |
|  | KEGG: Basal transcription factors | 0.026738 | 0.272729 | 9 | 2.076262 |
|  | KEGG: Sulfur relay system | 0.031615 | 0.293154 | 5 | 2.739512 |
|  | GO: photosynthesis | 6.21E-12 | 8.69E-11 | 52 | 2.879948 |
|  | GO: generation of precursor metabolites and energy | 3.70E-07 | 2.59E-06 | 55 | 2.056117 |
|  | GO: cellular homeostasis | 0.028619 | 0.133556 | 28 | 1.46484 |
|  | GO: peroxisome | 5.65E-08 | 4.52E-07 | 42 | 2.477904 |
|  | GO: chromatin binding | 0.018675 | 0.205423 | 7 | 2.550297 |
| Red | KEGG: Spliceosome | 1.75E-07 | 1.10E-05 | 22 | 3.493373 |
|  | KEGG: Sulfur relay system | 0.010058 | 0.316827 | 3 | 6.589773 |
|  | KEGG: Brassinosteroid biosynthesis | 0.020788 | 0.338545 | 2 | 8.786364 |
|  | KEGG: RNA transport | 0.025092 | 0.338545 | 9 | 2.12764 |
|  | KEGG: Ribosome biogenesis in eukaryotes | 0.026869 | 0.338545 | 6 | 2.614125 |
|  | KEGG: Aminoacyl-tRNA biosynthesis | 0.033747 | 0.354348 | 5 | 2.774641 |
|  | GO: endosome | 0.006888 | 0.048218 | 7 | 3.174742 |
|  | GO: cytoskeleton | 0.04369 | 0.152915 | 8 | 2.047777 |
|  | GO: receptor binding | 0.023548 | 0.193795 | 4 | 3.680116 |
|  | GO: chromatin binding | 0.041987 | 0.193795 | 3 | 3.886653 |
| Cyan | KEGG: Glycolysis / Gluconeogenesis | 0.007563 | 0.160406 | 4 | 4.973413 |
|  | KEGG: Carbon metabolism | 0.014179 | 0.160406 | 5 | 3.345062 |
|  | KEGG: Pyruvate metabolism | 0.014582 | 0.160406 | 3 | 5.730237 |
|  | KEGG: Purine metabolism | 0.041882 | 0.291603 | 3 | 3.820158 |
| Yellow | KEGG: Photosynthesis | 1.71E-06 | 0.000135 | 11 | 5.932757 |
|  | KEGG: Vitamin B6 metabolism | 0.000125 | 0.00492 | 4 | 13.80714 |
|  | KEGG: Fatty acid elongation | 0.000437 | 0.011498 | 6 | 5.917347 |
|  | KEGG: Pentose phosphate pathway | 0.002813 | 0.055561 | 8 | 3.287415 |
|  | KEGG: Biosynthesis of unsaturated fatty acids | 0.005292 | 0.083608 | 6 | 3.698342 |
|  | KEGG: Porphyrin and chlorophyll metabolism | 0.008038 | 0.105833 | 6 | 3.395199 |
|  | KEGG: Pantothenate and CoA biosynthesis | 0.011037 | 0.109053 | 5 | 3.672112 |
|  | KEGG: Carbon fixation in photosynthetic organisms | 0.011043 | 0.109053 | 9 | 2.446147 |
|  | KEGG: Plant hormone signal transduction | 0.013231 | 0.116136 | 16 | 1.834836 |
|  | KEGG: Carotenoid biosynthesis | 0.01945 | 0.140414 | 4 | 3.835317 |
|  | KEGG: Terpenoid backbone biosynthesis | 0.019735 | 0.140414 | 6 | 2.798745 |
|  | KEGG: Butanoate metabolism | 0.021329 | 0.140414 | 4 | 3.73166 |
|  | KEGG: Glyoxylate and dicarboxylate metabolism | 0.026625 | 0.161796 | 7 | 2.392327 |
|  | KEGG: Carbon metabolism | 0.035331 | 0.199366 | 18 | 1.576958 |
|  | KEGG: Glycolysis / Gluconeogenesis | 0.043062 | 0.226795 | 11 | 1.791021 |
|  | GO: photosynthesis | 8.24E-11 | 1.24E-09 | 33 | 3.773971 |
|  | GO: generation of precursor metabolites and energy | 8.53E-06 | 6.40E-05 | 31 | 2.393041 |
|  | GO: cellular homeostasis | 0.002697 | 0.013484 | 19 | 2.052528 |
| Black | KEGG: Valine, leucine and isoleucine degradation | 1.56E-08 | 1.20E-06 | 13 | 7.331226 |
|  | KEGG: Peroxisome | 1.16E-06 | 4.47E-05 | 14 | 4.726712 |
|  | KEGG: Galactose metabolism | 0.000639 | 0.013551 | 7 | 4.726712 |
|  | KEGG: Biosynthesis of unsaturated fatty acids | 0.000704 | 0.013551 | 6 | 5.49842 |
|  | KEGG: Fatty acid degradation | 0.001636 | 0.024317 | 7 | 4.036293 |
|  | KEGG: Fatty acid metabolism | 0.001895 | 0.024317 | 8 | 3.508963 |
|  | KEGG: Regulation of autophagy | 0.002957 | 0.032465 | 5 | 5.031234 |
|  | KEGG: Glyoxylate and dicarboxylate metabolism | 0.003373 | 0.032465 | 7 | 3.556734 |
|  | KEGG: Glycerolipid metabolism | 0.012059 | 0.103175 | 5 | 3.613985 |
|  | KEGG: Ether lipid metabolism | 0.016561 | 0.123743 | 3 | 5.49842 |
|  | KEGG: Pyruvate metabolism | 0.017678 | 0.123743 | 7 | 2.603117 |
|  | KEGG: alpha-Linolenic acid metabolism | 0.024467 | 0.152324 | 4 | 3.601304 |
|  | KEGG: Valine, leucine and isoleucine biosynthesis | 0.025717 | 0.152324 | 3 | 4.665326 |
|  | KEGG: Glycerophospholipid metabolism | 0.040217 | 0.221193 | 5 | 2.645288 |
|  | KEGG: Amino sugar and nucleotide sugar metabolism | 0.047417 | 0.243405 | 7 | 2.113118 |
|  | GO: response to extracellular stimulus | 0.003688 | 0.033188 | 13 | 2.380874 |
|  | GO: cell communication | 0.03009 | 0.135404 | 10 | 1.999324 |
|  | GO: peroxisome | 0.002768 | 0.002082 | 13 | 3.052269 |
|  | GO: lysosome | 0.09835 | 0.073948 | 1 | 35.33588 |
|  | GO: oxygen binding | 0.023701 | 0.097306 | 11 | 1.998346 |
|  | GO: carbohydrate binding | 0.04632 | 0.097306 | 8 | 2.024298 |
