# Supplementary material for: Comparison of gene co-networks reveals the molecular mechanisms of the rice (Oryza sativa L.) response to Rhizoctonia solani AG1 IA infection
Source: Funct Integr Genomics. 2018 May 5;18(5):545–57. doi: 10.1007/s10142-018-0607-y (PMC6097106; doi:10.1007/s10142-018-0607-y)
Supplement: Supplementary file 3 — (DOCX 24 kb) [file 10142_2018_607_MOESM3_ESM.docx]

| Table S2. Overview of the significantly overrepresented KEGG pathways and GO terms associated with the modules detected using WGCNA in Lemont | | | | | |
| --- | --- | --- | --- | --- | --- |
| WGCNA module | KEGG pathway or GO-term | pvalue | p.adjust | gene Count | Enrich.score |
| Yellow | KEGG: Biosynthesis of amino acids | 1.48E-07 | 1.23E-05 | 33 | 2.665984 |
|  | KEGG: Phenylalanine, tyrosine and tryptophan biosynthesis | 1.24E-05 | 0.000514 | 11 | 4.830304 |
|  | KEGG: Plant-pathogen interaction | 2.72E-05 | 0.000751 | 18 | 2.988148 |
|  | KEGG: Flavonoid biosynthesis | 0.001249 | 0.025911 | 5 | 5.918555 |
|  | KEGG: Stilbenoid, diarylheptanoid and gingerol biosynthesis | 0.006474 | 0.107467 | 4 | 5.185781 |
|  | KEGG: Riboflavin metabolism | 0.008408 | 0.111194 | 3 | 6.806338 |
|  | KEGG: Phenylpropanoid biosynthesis | 0.009378 | 0.111194 | 13 | 2.081939 |
|  | KEGG: Selenocompound metabolism | 0.014016 | 0.145411 | 4 | 4.188516 |
|  | KEGG: Fructose and mannose metabolism | 0.015979 | 0.147359 | 8 | 2.447223 |
|  | KEGG: Glycine, serine and threonine metabolism | 0.020355 | 0.159262 | 8 | 2.341966 |
|  | KEGG: Amino sugar and nucleotide sugar metabolism | 0.021993 | 0.159262 | 12 | 1.92179 |
|  | KEGG: mRNA surveillance pathway | 0.023857 | 0.159262 | 12 | 1.899443 |
|  | KEGG: Phenylalanine metabolism | 0.024945 | 0.159262 | 9 | 2.13068 |
|  | KEGG: Carbon metabolism | 0.030991 | 0.183731 | 22 | 1.520197 |
|  | KEGG: Fatty acid elongation | 0.038035 | 0.210459 | 4 | 3.111469 |
|  | GO: cell-cell signaling | 0.01029 | 0.154346 | 4 | 4.659219 |
|  | GO: Golgi apparatus | 3.59E-05 | 0.000251 | 27 | 2.374711 |
|  | GO: receptor binding | 0.00169 | 0.018593 | 7 | 4.050442 |
| Turquoise | KEGG: Photosynthesis | 2.57E-12 | 2.67E-10 | 27 | 4.522095 |
|  | KEGG: Photosynthesis - antenna proteins | 3.36E-08 | 1.74E-06 | 13 | 5.806146 |
|  | KEGG: Spliceosome | 1.22E-05 | 0.000422 | 55 | 1.775744 |
|  | KEGG: Circadian rhythm - plant | 0.001159 | 0.030141 | 14 | 2.460107 |
|  | KEGG: Fatty acid elongation | 0.013311 | 0.276879 | 8 | 2.450066 |
|  | KEGG: Porphyrin and chlorophyll metabolism | 0.023799 | 0.412523 | 11 | 1.932941 |
|  | KEGG: Basal transcription factors | 0.036047 | 0.535556 | 10 | 1.880533 |
|  | KEGG: Ribosome | 0.042157 | 0.548035 | 45 | 1.289724 |
|  | GO: photosynthesis | 4.25E-20 | 5.94E-19 | 75 | 3.254446 |
|  | GO: generation of precursor metabolites and energy | 1.97E-06 | 1.38E-05 | 63 | 1.845271 |
|  | GO: response to external stimulus | 0.017445 | 0.066791 | 21 | 1.640241 |
|  | GO: cellular homeostasis | 0.020374 | 0.066791 | 35 | 1.434613 |
|  | GO: tropism | 0.023854 | 0.066791 | 16 | 1.717155 |
|  | GO: nucleoplasm | 0.000804 | 0.00563 | 36 | 1.745914 |
|  | GO: nucleolus | 0.011389 | 0.039861 | 49 | 1.397025 |
|  | GO: peroxisome | 0.03735 | 0.087151 | 30 | 1.406592 |
|  | GO: chromatin binding | 0.000624 | 0.007487 | 11 | 3.100753 |
| Brown | KEGG: Diterpenoid biosynthesis | 2.18E-06 | 0.000189 | 9 | 7.39354 |
|  | KEGG: alpha-Linolenic acid metabolism | 8.27E-06 | 0.00033 | 10 | 5.62082 |
|  | KEGG: Oxidative phosphorylation | 1.14E-05 | 0.00033 | 16 | 3.487203 |
|  | KEGG: Valine, leucine and isoleucine degradation | 2.17E-05 | 0.000473 | 12 | 4.22488 |
|  | KEGG: Galactose metabolism | 0.000109 | 0.001902 | 10 | 4.215615 |
|  | KEGG: Arginine and proline metabolism | 0.004633 | 0.067182 | 9 | 2.799496 |
|  | KEGG: Caffeine metabolism | 0.005577 | 0.068593 | 2 | 16.01934 |
|  | KEGG: Fatty acid degradation | 0.006307 | 0.068593 | 8 | 2.879881 |
|  | KEGG: Phagosome | 0.009966 | 0.096337 | 9 | 2.485759 |
|  | KEGG: Pantothenate and CoA biosynthesis | 0.014883 | 0.12948 | 5 | 3.40837 |
|  | KEGG: Ascorbate and aldarate metabolism | 0.016408 | 0.129769 | 6 | 2.912607 |
|  | KEGG: Peroxisome | 0.02 | 0.144997 | 10 | 2.107808 |
|  | KEGG: Glycerolipid metabolism | 0.022779 | 0.152443 | 6 | 2.707494 |
|  | KEGG: Fatty acid metabolism | 0.029256 | 0.163336 | 8 | 2.190679 |
|  | KEGG: Biosynthesis of unsaturated fatty acids | 0.029646 | 0.163336 | 5 | 2.860596 |
|  | KEGG: Glycerophospholipid metabolism | 0.031256 | 0.163336 | 7 | 2.312069 |
|  | KEGG: Tryptophan metabolism | 0.03226 | 0.163336 | 4 | 3.286018 |
|  | KEGG: 2-Oxocarboxylic acid metabolism | 0.034223 | 0.163336 | 6 | 2.464513 |
|  | KEGG: Starch and sucrose metabolism | 0.035671 | 0.163336 | 14 | 1.699021 |
|  | KEGG: Lysine degradation | 0.044006 | 0.191425 | 4 | 2.980342 |
|  | GO: peroxisome | 0.01806 | 0.126421 | 14 | 1.88586 |
|  | GO: oxygen binding | 1.11E-07 | 9.95E-07 | 30 | 3.006915 |
| Magenta | KEGG: Protein processing in endoplasmic reticulum | 3.08E-11 | 1.45E-09 | 29 | 4.114779 |
|  | KEGG: Phagosome | 4.93E-08 | 1.16E-06 | 15 | 5.59605 |
|  | KEGG: Protein export | 3.38E-07 | 5.30E-06 | 12 | 6.256788 |
|  | KEGG: Endocytosis | 0.001559 | 0.018313 | 11 | 2.850523 |
|  | KEGG: mRNA surveillance pathway | 0.001974 | 0.018559 | 11 | 2.767659 |
|  | KEGG: N-Glycan biosynthesis | 0.002895 | 0.022679 | 6 | 4.188012 |
|  | KEGG: Plant-pathogen interaction | 0.004441 | 0.028501 | 10 | 2.638788 |
|  | KEGG: Citrate cycle (TCA cycle) | 0.004851 | 0.028501 | 7 | 3.328932 |
|  | KEGG: Sphingolipid metabolism | 0.008193 | 0.042785 | 4 | 4.945842 |
|  | KEGG: Proteasome | 0.018256 | 0.085805 | 6 | 2.853371 |
|  | KEGG: Pyruvate metabolism | 0.039988 | 0.170857 | 7 | 2.195165 |
|  | GO: Golgi apparatus | 6.24E-16 | 4.37E-15 | 33 | 5.779536 |
|  | GO: nuclear envelope | 0.000587 | 0.002056 | 7 | 4.91451 |
|  | GO: endosome | 0.002634 | 0.006147 | 7 | 3.793078 |
|  | GO: cytoskeleton | 0.005911 | 0.010345 | 9 | 2.752445 |
|  | GO: nucleolus | 0.009298 | 0.013017 | 13 | 2.120402 |
|  | GO: enzyme regulator activity | 0.001405 | 0.012641 | 13 | 2.662492 |
| Tan | KEGG: Proteasome | 2.53E-08 | 1.26E-06 | 11 | 8.986898 |
|  | KEGG: Oxidative phosphorylation | 2.32E-05 | 0.00058 | 10 | 5.057561 |
|  | KEGG: Phagosome | 0.000147 | 0.002448 | 8 | 5.127321 |
|  | KEGG: Endocytosis | 0.00687 | 0.072886 | 7 | 3.116306 |
|  | KEGG: Protein processing in endoplasmic reticulum | 0.007289 | 0.072886 | 10 | 2.437579 |
|  | KEGG: Protein export | 0.024996 | 0.208298 | 4 | 3.582947 |
|  | KEGG: Homologous recombination | 0.046362 | 0.289761 | 3 | 3.717308 |
|  | KEGG: SNARE interactions in vesicular transport | 0.046362 | 0.289761 | 3 | 3.717308 |
|  | GO: endosome | 0.000363 | 0.002544 | 6 | 6.427679 |
|  | GO: nuclear envelope | 0.000802 | 0.002808 | 5 | 6.94003 |
|  | GO: translation factor activity, RNA binding | 0.027247 | 0.095786 | 6 | 3.535814 |
|  | GO: enzyme regulator activity | 0.038314 | 0.095786 | 4 | 2.43195 |
| Midnightblue | KEGG: Sulfur metabolism | 0.013023 | 0.201855 | 2 | 11.37059 |
|  | KEGG: Regulation of autophagy | 0.013023 | 0.201855 | 2 | 11.37059 |
|  | KEGG: Fructose and mannose metabolism | 0.036997 | 0.271633 | 2 | 6.51573 |
|  | KEGG: Valine, leucine and isoleucine degradation | 0.03853 | 0.271633 | 2 | 6.372527 |
|  | KEGG: Sesquiterpenoid and triterpenoid biosynthesis | 0.043964 | 0.271633 | 1 | 22.30385 |
